# Protein-Carbohydrate Interaction between Sperm and the Egg-Coating Envelope and Its Regulation by Dicalcin, a *Xenopus laevis* Zona Pellucida Protein-Associated Protein

**DOI:** 10.3390/molecules20059468

**Published:** 2015-05-22

**Authors:** Naofumi Miwa

**Affiliations:** Department of Physiology, Toho University, Tokyo 143-8540, Japan; E-Mail: nmiwa@med.toho-u.ac.jp; Tel.: +81-3-3762-4151; Fax: +81-3-3762-8225

**Keywords:** egg-coating envelope, sperm, ZP protein

## Abstract

Protein-carbohydrate interaction regulates multiple important processes during fertilization, an essential biological event where individual gametes undergo intercellular recognition to fuse and generate a zygote. In the mammalian female reproductive tract, sperm temporarily adhere to the oviductal epithelium via the complementary interaction between carbohydrate-binding proteins on the sperm membrane and carbohydrates on the oviductal cells. After detachment from the oviductal epithelium at the appropriate time point following ovulation, sperm migrate and occasionally bind to the extracellular matrix, called the zona pellucida (ZP), which surrounds the egg, thereafter undergoing the exocytotic acrosomal reaction to penetrate the envelope and to reach the egg plasma membrane. This sperm-ZP interaction also involves the direct interaction between sperm carbohydrate-binding proteins and carbohydrates within the ZP, most of which have been conserved across divergent species from mammals to amphibians and echinoderms. This review focuses on the carbohydrate-mediated interaction of sperm with the female reproductive tract, mainly the interaction between sperm and the ZP, and introduces the fertilization-suppressive action of dicalcin, a *Xenopus laevis* ZP protein-associated protein. The action of dicalcin correlates significantly with a dicalcin-dependent change in the lectin-staining pattern within the ZP, suggesting a unique role of dicalcin as an inherent protein that is capable of regulating the affinity between the lectin and oligosaccharides attached on its target glycoprotein.

## 1. Introduction

Much attention has been paid to gaining greater understanding of the molecular mechanisms of the fertilization process whereby two gametes (*i.e.*, sperm and egg) interact, achieve mutual recognition, and fuse to initiate the resumption of the cell cycle of eggs, which ultimately leads to development of a new individual. The hypothesis that fertilization success involves carbohydrate recognition between the sperm and egg was initially proposed about fifty years ago [1,2]. Since then, research interest in this field has grown markedly, as evidenced by the hundreds of research papers that are identified upon a literature search using five search terms (*i.e*., sperm, egg, interaction, protein, carbohydrate). Evidence has accumulated showing that, on the route of the sperm toward the egg, there are at least two regulatory steps that involve protein-oligosaccharide interactions: one is the sperm-oviductal epithelium interaction, and the other is the sperm-zona pellucida (ZP) interaction. In both these interactions, sperm recognition of the target involves the binding of multiple carbohydrate receptor proteins on the sperm to the complementary oligosaccharide chains attached to the surface of the target cells. Many candidates and combinations of interacting proteins and carbohydrates have been reported across divergent species and have been discussed in numerous excellent reviews [3,4,5,6,7,8,9,10,11]. In addition to these molecules, we have recently isolated and characterized dicalcin, a unique glycoprotein-associated protein, which regulates the affinity of interaction between a lectin and the carbohydrate moieties of glycoprotein, affecting fertilization success in *Xenopus laevis* [12]. Dicalcin, a non-enzyme protein in the frog ZP, binds to the glycoprotein that constitutes the filamentous ZP, inducing alterations in the distribution pattern of the oligosaccharides within the ZP, which suppresses the ability of sperm to penetrate the ZP. This finding demonstrates a unique aspect of protein-carbohydrate interaction because the properties (e.g., clustering or motility) of oligosaccharides attached on the glycoprotein may be regulated by the binding of its associated protein. It should be noted that, contrary to the significant action of carbohydrates on sperm-ZP interaction, the polypeptide moiety of a constitutive ZP protein and/or a three dimensional structure of the ZP have been reported to be important for sperm binding in mice [13], and it remains crucial that the currently proposed mechanisms for sperm-ZP interaction are integrated in future. In this review, to consolidate the current knowledge on protein-carbohydrate interaction in the communication between sperm and cells of the female reproductive system (*i.e*., oviductal epithelial cells, and eggs), we briefly describe the fertilization process, sperm storage in the oviduct, sperm interaction with the egg-coating envelope, and the molecules involved with these sperm behaviors, followed by a review of the unique action of dicalcin.

## 2. Brief Introduction to Fertilization

Fertilization is a process whereby sperm recognize the egg-coating envelope, undergo the acrosome reaction, penetrate the egg-coating envelope in a species-restricted manner, and then fuse with the egg, thereby initiating the resumption of the cell cycle of the egg, which leads to generation of a new organism harboring hereditary information from the parents. Details of these sequentially and highly coordinated events are beyond the scope of this review, but are excellently summarized elsewhere [14]. Briefly, for example in mammals, sperm are produced in the testes (e.g., at the rate of *ca.* 1000 sperm/s), and then, male reproductive proteins such as spermadhesin [15] and CRISP [16] facilitate maturation of the sperm in the epididymis [17]. In eutherian animals, great numbers of sperm are deposited in the female reproductive tract upon ejaculation, although only a few thousand enter the oviduct and even fewer reach the ampulla region of the oviduct at the appropriate time window for fertilization, and only one spermatozoon actually fertilizes the egg. Sperm in the female reproductive tract remain unable to fertilize eggs until the sperm membrane undergoes various physiological changes, called capacitation which renders sperm ready for fertilization (for reviews see [18,19]). Glucose, cholesterol, bicarbonate, intracellular Ca^2+^, and many other factors are considered to be involved in capacitation; however, the precise molecular mechanisms underlying their action are yet to be clarified. Once capacitated, sperm are competent for the progesterone-dependent activation of the sperm Ca^2+^ channel, catsper [20]. This leads to a vigorous swimming pattern called hyperactivation, a movement considered to confer to the sperm the strong thrusting power that facilitates their movement to the cumulus cell layer and the extracellular matrix, ZP: both structures surround the oocytes. In a species-restricted interaction, sperm then undergo an exocytotic event called the acrosome reaction, during which a variety of lytic enzymes as well as ZP-binding proteins are released, which facilitates sperm penetration of the ZP. Ultimately, sperm reach the plasma membrane of oocytes and fuse with them, initiating the resumption of the arrested cell cycle.

## 3. Sperm Storage in the Oviduct

Before fertilization takes place *in vivo*, mammalian sperm are stored in the caudal part of the isthmus of the oviduct through binding to the oviductal lumen (for reviews see [21,22]). This adhesion is considered to involve seminal plasma proteins (e.g*.*, spermadhesin) that coat the surface of sperm: the adhesion establishes the sperm reservoir and prevents premature occurrence of capacitation and the acrosome reaction. With hormonally changing conditions around the time of ovulation, sperm may shed their protein coat, thereby creating new surface structures that allow sperm to be released from the epithelium, to complete capacitation and prepare them to interact with the ZP in the appropriate manner, leading to fertilization. The following interactions between protein-carbohydrate in this process have been studied.

### Spermadhesin and Oligosaccharides of the Oviductal Epithelial Cells

Spermadhesin (12–14 kDa) is secreted from the male reproductive tract into the seminal plasma of mammalian species, including pigs, cattle, and horses, and is a major constituent of the seminal plasma proteins (e.g., *ca.* 80% of these proteins in pigs; for a review see [15]). Once secreted, it binds to the lipids on the sperm surface by forming a heterodimer with a distinct phosphocholine-binding protein, pB1 [23]. Subsequently, spermadhesin on the sperm surface serves attachment of sperm to the oviductal lumen in the pig, where sperm wait to interact with the oocyte at the appropriate timing, for hours or even days. Spermadhesins comprise a group of 110-133-residue polypeptides, encoded by five closely related genes (*AQN1*, *AQN3*, *AWN*, *PSP1*, *PSPII*). Due to their ZP-binding capability, they are thought to play a role during the initial sperm-ZP attachment. AQN-1 shows a broad carbohydrate-binding pattern: it recognizes both α- and β-linked galactose as well as mannose [24]. AWN and AQN-3 show equivalent binding affinity for Galβ1-3GalNAc and Galβ1-4GlcNAc sequences [25,26]. The underlying molecular basis of these isoform-distinct carbohydrate-binding patterns remains unknown.

## 4. Sperm Recognition of the ZP

### 4.1. Structure of the ZP

After a certain stage of development, the oocytes of animals are coated with an extracellular envelope that is called either the zona pellucida (ZP) in mammals, the vitelline envelope (VE) in nonmammals, or the chorion in fish (for reviews see [14,27,28]). Since the gross structure and function of these egg coats are similar, we will refer to them collectively as the ZP in this review, regardless of their different names. This extracellular matrix with a thickness of *ca.* 2–25 μm plays multiple roles in the generation and development of the zygote, including species-restricted recognition between gametes, induction of the acrosomal reaction, prevention of polyspermy by its structural modification following fertilization and protection of the fragile preimplantation embryo from physical damage [29]. The ZP contains three-dimensional filaments formed by polymerization of ZP proteins (see below) and other components, including hyaluronic acid or ZP protein-associated proteins. Within the mature unfertilized ZP, individual filaments (*ca.* 5–20 nm diameter for *Xenopus laevis*) [30] are uniformly distributed in a randomly oriented array, which confers the competence for fertilization to the ZP. The fine ZP structure, following fertilization success, is converted to a sperm-impenetrable (*i.e*., incompetent) status (called the fertilization envelope, FE) to block polyspermy, mediated by fusion-triggered release of the cortical granule contents. The FE is multilayered with fibrous sheets that twist and curl and sometimes merge, and are elevated away from the egg surface, widening the space between the envelope and the egg surface [31].

### 4.2. ZP Proteins of the Extracellular Egg-Coating Envelope

The ZP filaments are composed of only three to four glycoproteins, including ZP1-4 in humans, ZP1-3 in mice, and gp120, gp69/64, gp41, and gp37in *Xenopus laevis* [32]. Early studies on mammalian species identified protein components of the ZP based on SDS-PAGE; these ZP proteins are termed ZP1, ZP2, and ZP3 from higher to lower molecular weight [33,34]. Subsequently, more ZP proteins have been identified; the amino acid sequences of these have been found to be highly conserved during evolution in mammals and across widely divergent species, such as amphibians and marine invertebrates [35,36,37,38]. Due to the history of their identification in the egg, the name of each ZP protein varies depending on its origin, and the names are still used interchangeably (e.g., ZPA or ZP2 or gp64/69, ZPB or ZP1 or gp37, ZPC or ZP3 or gp41). To prevent possible confusion, we here refer to ZPA, ZPB and ZPC throughout this review, as also suggested by others [39]. ZP proteins are mainly synthesized in growing oocytes during each reproductive cycle in mammalian and amphibian species, whereas fish and bird ZP proteins are synthesized in the ovary or liver or both (for a review see [32]). Post-translational modifications and processing of the protein backbone are species-specific events, resulting in heterogeneity of the attached oligosaccharides and in distinct polypeptide chain lengths (for a review see [38]). ZP proteins carry a complex pattern of *N*- and *O*-linked oligosaccharides. In the mouse ZP, ZPA, ZPB and ZPC possess four, six, and five *N*-linked carbohydrates, respectively. ZPC contains at least two sites of O-linked carbohydrates [40,41].

ZP protein binds to different ZP protein isoforms via a conserved motif called the ZP domain, spanning *ca.* 260 amino acids [42], which generates μm-long filaments, and pairs of which are interconnected to form the three-dimensional meshwork [43]. A recent X-ray crystallographic study on ZP proteins has demonstrated that this ZP domain is divided into two domains (*i.e*., N-terminal ZP-N and C-terminal ZP-C), each of which is considered to function in the dimerization of ZP proteins [44,45]. In particular, ZP-C mediates the specific interactions between different ZP proteins during polymerization [45]. In addition, it has been reported that there is a potential sperm-binding region within the ZP-C domain [46] and/or nearby the ZP-C domain [47]. It should be noted that ZP proteins have now been reported in differentiated epithelial cells and neural tissues other than eggs, and they have been suggested to involved in the formation of extracellular matrices as well as in remodeling of the intracellular cytoskeleton during morphological differentiation (for a review see [38]).

### 4.3. Carbohydrates of the ZP

Carbohydrates within the unfertilized ZP have been characterized by many methodological approaches including lectin-based cytochemistry, and biochemical and mass spectrometric analyses of enzymatically and chemically released glycans. Comparative cytochemical studies of the mammalian unfertilized ZP, using a set of lectins, have demonstrated that variations in the staining pattern correlated with the evolutionary distance between species: there is a highly similar pattern among phylogenetically closely related species, such as the rodents and rabbits, whereas there are also species-specific staining patterns [48]. Some lectins such as common lentil agglutinin (LCA), *Ricinus communis* agglutinin I (RCA-I), and wheat germ agglutinin (WGA) reacted with the unfertilized ZP of almost all mammalian species, indicating that βGal, GlcNAc and mannose are commonly present in most mammalian ZP, including the human ZP [49]. However, there is still a species-preferential staining pattern. For example, in mice and rats, *Griffonia simplicifolia* (GS-I) and soybean agglutinin (SBA) showed an intense staining of the ZP, indicating that αGal and GalNAc are present in the ZP of these animals [48]. Additionally, in the frog, RCA-I preferentially reacted with the ZP, while SBA and *Sambucus nigra* (SNA) exhibited no binding, which indicated the presence of Gal, but absence of GalNAc and sialic acid in the frog ZP [12]. Furthermore, our previous study of the frog ZP has shown that RCA-I reacts with the outermost layer of the VE, indicating that the distribution pattern of oligosaccharides is not homogeneous throughout the ZP.

Comparison of mass spectrometric analyses of unfertilized ZP among a variety of species revealed a few conserved carbohydrate components. In the human ZP, the sialyl-Lewis^X^ sequence [NeuAcα2-3Galβ1-4(Fucα1-3)GlcNAc] is the most abundant terminal sequence on both the *N*- and *O*-glycans [50]. Terminal sialyl-Lewis^X^ is expressed in *ca.* 85% of all N-glycans, with varied configurations of bi-, tri-, and tetra-antennae (for a review see [51]), while the high mannose-type *N*-glycan is absent, suggesting that the sperm membrane protein required for binding to the egg may be a C-type lectin with a binding specificity that overlaps with that of the selectins of leukocytes [50]. This peculiar profile is similar to that of the porcine ZP; however, it is in sharp contrast to the profile of *N*- and *O*-glycans of the bovine and murine ZP [52,53]. Analysis of murine and bovine ZP glycosylation reveals substantial amounts of high mannose-type *N*-glycans and a greater structural diversity of complex type *N*- and *O*-glycans as compared to human ZP glycans. The degree of acidity (*i.e*., sulfation and sialylation) on both *N*- and *O*-glycans in the ZP proteins is highly heterogeneous across species. For example, mouse and bovine ZP proteins contain mainly acidic tri- and tetra-antennary chains, and the sulfate contents in both species are lower than that in the pig ZP; therefore, the acidic properties of the ZP of these two species are mainly due to their sialic acid content. In porcine and bovine species, neutral oligosaccharides constitute *ca.* 25% of the total carbohydrates, while the content of neutral *N*-glycans is less than 5% in mice. In the frog ZP, the *N*-glycans of the ZP are composed of a heterogeneous mixture of high mannose and neutral (from ZPC)- and acidic (from ZPA)-complex oligosaccharides. One of these complexes possesses terminal GlcNAc residues. Furthermore, the *O*-glycan is thought to be the trisaccharide GalNAc-βGal-βGalNAc [54]. The above difference in carbohydrates among species may underlie species-restricted sperm recognition. A concised summary of the lectin-staining pattern and composition of carbohydrates within the ZP is shown in Table 1.

**Table 1:** Carbohydrates of the egg-coating envelope of human, mouse and frog.

	*N*-Linked Glycan	*O*-Linked Glycan	Major Terminal Residues	Lectin-Staining Intensity
Human	NeuAcα2-3Galβ1-4(Fucα1-3)GlcNAc	NeuAcα2-3Galβ1-4(Fucα1-3)GlcNAc	NeuAc from sialyl-Lewis^X^	WGA > ConA, MPA
Mouse	High-mannose type Complex type	Core type-1 (Minor) Core type-2 (Major)	GlcNAc Gal from LacNAc NeuAc from Sd1 antigen	GS-I, DBA > WGA, RCA-I, PNA
Frog	High-mannose type Complex type	Trisaccharides (GalNAc-βGal-βGal)	GlcNAc	WGA > ConA = MPA

### 4.4. Carbohydrate Structures Determining Gamete Recognition

Carbohydrate recognition by sperm has been thought to play a critical role in the sperm-ZP interaction, and much evidence in this regard has been demonstrated particularly in mice; however, the underlying mechanisms have not yet been fully integrated (for reviews see [7,8,9]. In the case of mice, there is a consensus that certain O-linked oligosaccharides are critical for achieving high-affinity sperm binding. In contrast, the identity of the sugar moieties that determine the specific terminal carbohydrate for sperm-ZP interaction (*i.e*., α/βGal, GlcNAc or fucose) is still debated. The degree of sialylation and sulfation appears to have no effect on sperm-binding activity. Additionally, recent findings of abundant expression of sialyl-Lewis^X^ in the human ZP suggests that a C-type lectin such as the selectins of leukocytes is required for sperm binding to the egg, although selectin itself is absent from human sperm; the receptor protein(s) for this ligand therefore remains unknown [55]. In *Xenopus laevis*, the *N*-glycan of ZPC (with GlcNAc as its terminal residue) [56] and/or *O*-glycan of ZPA (with an as-yet-unidentified terminal residue) [57] are suggested to be responsible for sperm binding. In addition, heteromeric ZPA/ZPB/ZPC complexes facilitate sperm binding to the ZP [56].

### 4.5. Interactive Proteins with Carbohydrates in the ZP

Many different sperm molecules have been shown to recognize oligosaccharides of the ZP. They include fucosyltransferase 5 (FUT5), β1,4-galactosyltransferase (GalT), ZP3R(sp56), and pro/acrosin (for reviews see [7,8,55]. Catalytic enzymes such as FUT5 and GalT, present on the sperm surface, do not perform their catalytic action because of a lack of substrates, and consequently, their function is confined to their carbohydrate-binding action via carbohydrate-lectin-like interactions. Some of these are briefly summarized below. It should be noted that virtually none of the proteins reported have been shown to be indispensable for fertilization when the genes encoding these proteins were disrupted by homologous recombination in mice. It is now assumed that the binding of sperm to the ZP involves multiple sperm proteins, providing an element of redundancy to a process that is very crucial to the maintenance of the species during evolution.

#### 4.5.1. FUT5 and *N*-Acetyllactosamines

Mammalian sperm express fucosyltransferase activities, whereas FUT5 is found only in humans and chimpanzees [58]. FUT5 is thought to recognize *N*-acetyllactosamines (Galβ1-3GlcNAc, Galβ1-4GlcNAc) as well as GalNAcβ1-4GlcNAc, regardless of their modification by sulfation, sialylation, or fucosylation [59,60]. FUT5 is an integral membrane protein with an intracellularly oriented N-terminus and extracellularly oriented C-terminus. It apparently mediates adhesion of sperm to the ZP via recognition of the *N*-acetyllactosamine terminus of the ZP. The anti-FUT antibody and FUT substrates blocked sperm binding to the ZP, whereas they failed to induce the acrosome reaction *in vitro*, suggesting that FUT5 is related to the binding of the sperm to the ZP, rather than to the acrosome reaction [61].

#### 4.5.2. Galactosyltransferase (GalT) and *N*-Acetylgulucosamine on ZPC

β1,4-galactosyltransferase (GalT), responsible for galactosyltransferase activities on sperm surface [62] recognizes the terminal *N*-acetylglucosamine residues on ZPC oligosaccharides. Sperm devoid of GalT are able to bind to the ZP, but do not respond to the ZPC-induced acrosome reaction [63], suggesting that GalT is dispensable for mouse sperm-ZP binding, but is important for the acrosome reaction. It has been considered that the GalT-ZP interaction causes aggregation of the sperm surface GalT and triggers pertussis toxin-sensitive signaling pathways that contribute to induction of the acrosome reaction [64,65].

#### 4.5.3. ZP3 Receptor (ZP3R, Formerly Called sp56) and *O*-Linked Oligosaccharide on ZPC

ZP3R was initially isolated by ZPC-affinity chromatography in mice [66], and was also found in guinea pigs [AM67, [67]], and rats [68], but no sequence data are available for a human orthologue. ZP3R is a component of the acrosomal matrix; following maturation, ZP3R becomes transiently associated with the sperm surface [69]. After the acrosome reaction occurs, ZP3R is released into the surrounding environment and interacts with an *O*-linked oligosaccharide (*ca.* 3.9 kDa) of ZPC [66]. Both *in vivo* and *in vitro* fertilization of homozygous ZP3R-null mice were normal, as compared with wild type littermates, suggesting that ZP3R is dispensable for mouse fertilization [70].

#### 4.5.4. Pro/Acrosin and Polysaccharide of the ZP

Acrosin is a serine protease of mammalian sperm, localized in the acrosome, as the zymogen form, proacrosin, which is autoactivated *in vitro* by processing both at the N- and C-terminal ends at a basic pH (for reviews see [71,72]). Pro/acrosin is characterized by its high affinity binding (Kd: 10^−8^ M) to sulfated polysaccharides, such as fucoidan and the ZP proteins; however, this binding does not correlate with its proteolytic activity [73,74]. Because of its ZP-binding activity, acrosin has long been believed to be involved in various aspects of fertilization, including the recognition and binding between gametes, and sperm penetration of the ZP. However, the sperm of homozygous null mice are fertile, and are able to penetrate the ZP, indicating that acrosin is not essential for either sperm penetration of the ZP or fertilization [75]. In terms of the serine protease activity and the fertilization process, mouse sperm possess at least two serine proteases, acrosin and PRSS21 (testisin/TESP5), both of which are localized to the acrosome. Single knockout of these two genes showed that they are not essential for fertilization. However, double-knock out impairs fertility *in vivo*, and to an even greater degree *in vitro*, which suggests that sperm trypsin-like activity is indispensable for *in vitro* fertilization but not particularly for fertilization *in vivo* in mice [76].

## 5. Dicalcin as An Inherent Suppressive Factor for Fertilization in *Xenopus laevis*

We have recently isolated and characterized dicalcin, a unique glycoprotein-associated protein, present in the frog ZP [12]. The primary structure of dicalcin shows no enzymatic motif, and therefore dicalcin is thought to function by binding to its target protein. Indeed, dicalcin binds to ZPC (gp41), and induces alteration in the distribution pattern of oligosaccharides of ZPC, affecting the fertilization process.

### 5.1. Structure and Distribution of Dicalcin

Dicalcin is an S100-like Ca^2+^-binding protein, present in the frog ZP [12,77]. The S100 protein family comprises small (10–14 kDa) calcium binding proteins that regulate various extra- and intracellular activities (for reviews see [78,79]). The primary structure of dicalcin consists of two S100-like regions connected by a linker region, which features this protein as a “dimeric form of S100 calcium binding protein”. Since S100 proteins are known to function as dimers, it is rational to consider that dicalcin in its monomeric form may exert function(s) similar to those of other S100 members. Indeed, its three-dimensional structure is reasonably represented by the folding pattern of the dimeric form of an S100 protein [80]. Extensive biochemical analysis has revealed that the Ca^2+^ binding mechanism of dicalcin: (1) four Ca^2+^-binding motifs (called EF-hands) in the dicalcin sequence are functional; therefore dicalcin is capable of binding to ~four Ca^2+^ per protein; and (2) the first and second Ca^2+^ binding to the higher-affinity EF-hands induce major conformational change accompanied by an increase in the α-helical content, as measured with circular dichroism. This conformational change may represent the molecular basis for Ca^2+^-dependent binding of dicalcin to target molecules [81]. Dicalcin was originally identified in frog (*Rana catesbeiana*) olfactory cilia as an intracellularly expressed Ca^2+^-binding protein [82]. After its original identification, however, this protein was also found in other tissues, including egg and lung [83]. In *Xenopus laevis* eggs, dicalcin is localized uniformly in the extracellular ZP as well as in the intracellular marginal region of an egg, which suggests that dicalcin would be released from the egg and retained within the ZP. Dicalcin lacks an N-terminal leader sequence and therefore the secretion pathway of dicalcin may be different from the classic ER-Golgi pathway. It should be noted that N-terminal leaderless secretion has been observed for other proteins, including interleukin 1β and fibroblast growth factor-2 [84].

### 5.2. Binding of Dicalcin to Target Proteins

Dicalcin shows no enzymatic activities in and of itself, and instead, through interactions with target proteins, it serves to regulate cellular events. In *Xenopus* eggs, dicalcin interacts with several egg proteins, including ZPC (gp41) and ZPB (gp37) of the ZP [12]. The binding of dicalcin to ZPC and ZPB is mediated via interaction between the protein cores of two proteins, but not via dicalcin and a carbohydrate of the ZP protein. The external Ca^2+^ concentration is high, so that dicalcin is retained with ZP proteins in the ZP, as confirmed by the immunohistochemical study described above. Through this interaction with ZP proteins, dicalcin plays a role particularly in fertilization (see below). In addition to the egg, dicalcin also binds to some ciliary proteins, including annexins and a β-adrenergic receptor kinase-like protein [85,86], serving to regulate the ciliary function(s) of olfactory neurons such as chemosensory signaling and/or ciliary membrane repair (for a review see [87]).

### 5.3. Effects of Dicalcin on Fertilization Success

The amount of dicalcin in the ZP substantially affects the fertilization rate in *Xenopus laevis*: preincubation of unfertilized *Xenopus* eggs with a dicalcin-specific antibody increases the fertilization rate, whereas preincubation with recombinant dicalcin at μM levels inhibits fertilization as well as sperm binding to the ZP and *in vitro* sperm penetration through the ZP protein layer. It should be noted that this suppressive action occurs in unfertilized eggs at fertilization, which precedes the polyspermy block observed after fertilization. Dicalcin treatment reduced sperm binding to the ZP only to *ca.* 77% of the control value, whereas it inhibited sperm penetration *in vitro* to *ca.* 50% of the control, implying that dicalcin preferentially affects the sperm penetration process, rather than the initial sperm-ZP binding. Through these actions, dicalcin inherently suppresses fertilization [12]. It should be noted that *Xenopus laevis* egg-coating extracellular structures include an outer and inner coat called the egg jelly and ZP, respectively, and the outer egg jelly was removed in our previous *in vitro* fertilization experiments as described above.

## 6. Regulation of Oligosaccharide-Lectin Interaction by Dicalcin through Its Binding to ZPC

### 6.1. Sperm Binding to Xenopus Laevis ZP

*Xenopus laevis* ZP is composed of at least four ZP proteins [ZPA(gp64/69), ZPB(gp37), ZPC(gp41), ZPX(p120)]. It should be remembered that this nomenclature of ZP proteins is based on a previous report [39]. These ZP proteins contain four (ZPA), zero (ZPB), two (ZPC), or fourteen (ZPX) potential N-linked glycosylation sites. The frog ZP remains fertilization incompetent until the passage of the pars recta, the anteriormost part of the frog oviduct, where oviductin, a serine protease secreted from the oviduct, processes gp43 (premature ZPC) to gp41 (mature ZPC) [88], concomitant with some structural alterations of the ZP, ultimately conferring fertilization competence to the ZP. A solid binding assay using ZP protein-coupled glass slides showed that most sperm binding activity (*ca.* 70%) is ascribed to ZPC and the remainder to ZPA (gp64/69) [54]. In contrast, a competitive assay for examination of sperm-binding to eggs *in vitro* using ZP proteins revealed that ZPA and its *N*-glycan have the most inhibitory activity, suggesting that ZPA and its *N*-glycans mostly mediate sperm-VE interaction [57,89]. Although the reason for the above inconsistent results remains unknown, it may be that sperm may utilize these ZP proteins in the multiple processes of sperm binding described earlier. 

### 6.2. Regulation of Oligosaccharide-Lectin Interaction by Dicalcin

**Figure 1:**
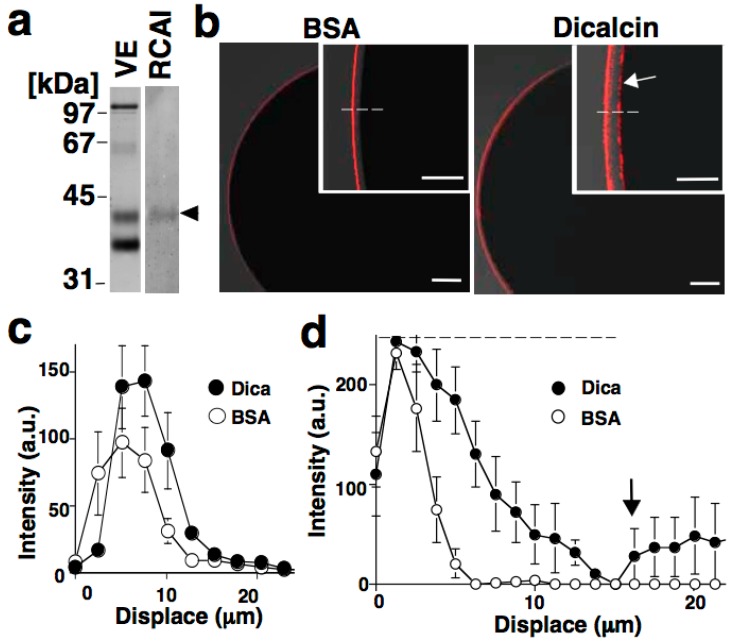
Dicalcin-mediated change in RCA-I reactivity of the zona pellucida (ZP). (**a**) Blots of the vitelline envelope (VE) were treated with rhodamine-labeled RCA-I. RCA-I recognizes ZPC (*arrowhead*); (**b**) Representative confocal images of a *Xenopus laevis* egg treated with rhodamine-labeled RCA-I. Unfertilized eggs were pretreated with bovine serum albumin (BSA) or dicalcin, followed by RCA-I staining. Insets: higher magnification images. The egg plasma membrane is indicated (*arrow*). Scale bar: 50 μm; (**c**) Averaged line scans of RCA-I staining across the frog ZP under lower-sensitivity detection. The position where the RCA-I signal starts to increase is designated as 0 μm in the x-axis; (**d**) Averaged line scans of RCA-I staining across the frog ZP under higher sensitivity detection. An increase in RCA-I signal at the interface between VE and egg plasma membrane is indicated (*arrow*). Reproduced from Miwa *et al*., (2010) [12] with permission of the publisher.

**Figure 2:**
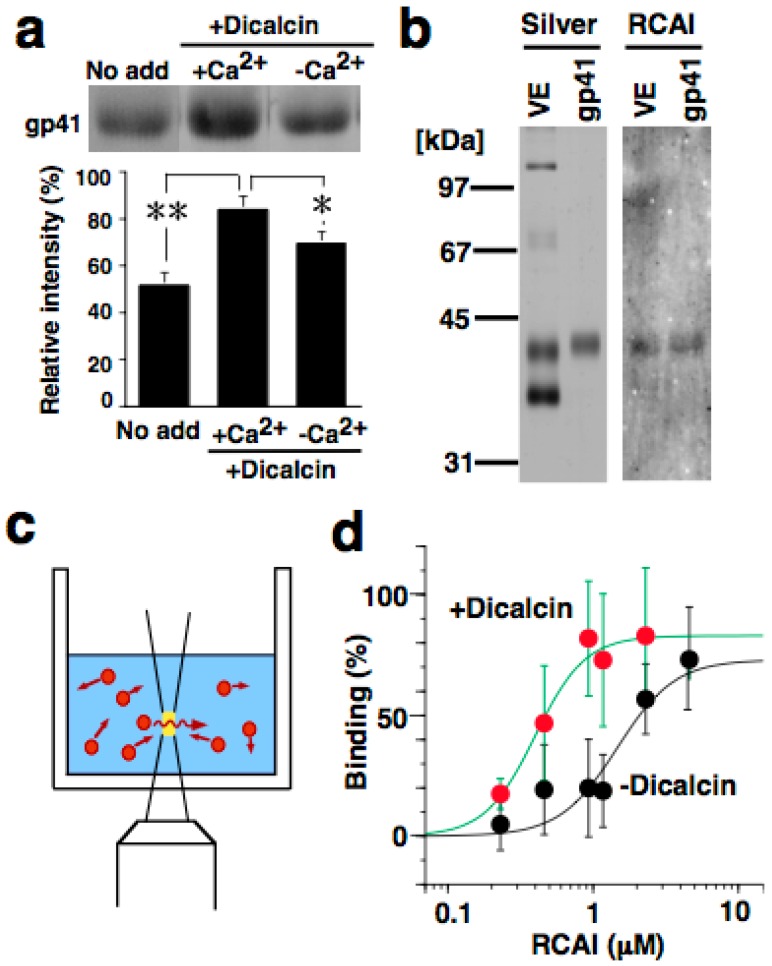
Dicalcin-binding to frog ZPC increases the RCA-I reactivity of ZPC. (**a**) Blots of frog ZP proteins were preincubated in the presence or absence of dicalcin, followed by incubation with rhodamine-labeled RCA-I (upper). The normalized intensity is also shown (lower, *n* = 8; *, *p* = 0.06; **, *p* = 0.002); (**b**) Isolation of frog ZPC. Silver, silver-stained frog ZP proteins and isolated ZPC; RCA-I, blot with RCA-I; (**c**) Schematic representation of the fluorescent signal measurement. A fluorescent signal of the diffusing molecule (red) was detected within a small defined confocal volume (yellow) of the well. Autocorrelation analysis of the fluctuating fluorescent signal estimates the diffusion time of fluorescent molecules and distinguishes small fast-diffusing (*i.e*., free fluorescent molecules) and large slow-moving (target-bound molecules) molecules; this enabled us to investigate the stoichiometry of the binding; (**d**) The normalized binding activity of isolated fluorescently labeled ZPC to RCA-I. Reproduced from Miwa *et al*., (2010) [12] with permission of the publisher.

**Figure 3:**
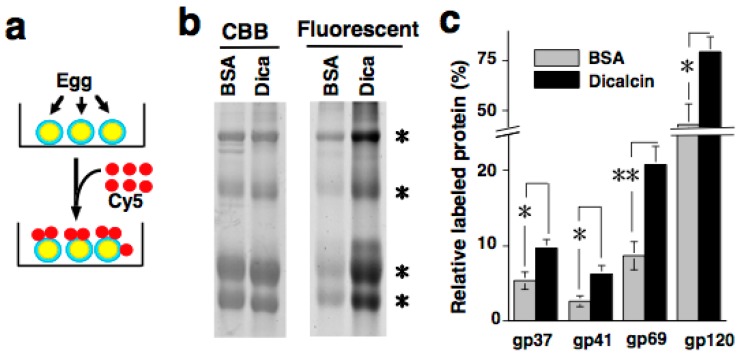
Dicalcin-binding to frog ZPC changes the three-dimensional structure of the entire ZP (**a**) Schematic representation of *in vivo* labeling of the frog ZP with fluorescent dye; (**b**) After preincubation either with bovine serum albumin (BSA) or dicalcin, labeled ZP proteins were electrophoresed and fluorescent images were obtained, followed by quantification of the amount of each ZP protein (*asterisks*); (**c**) The ratio of the molar amount of labeled protein to the total amount existing in the preparation was calculated for each frog ZP protein (*n* = 8–10; *, *p* < 0.02; **, *p* = 0.002). Reproduced from Miwa *et al*., (2010) [12] with permission of the publisher.

Quantification of RCA-I immunosignals revealed that pretreatment with dicalcin increased the intensity of the RCA-I signal in the outermost region of the ZP, and broadened the RCA-I reactivity within the ZP of both (*i.e*., animal and vegetal) hemispheres [12]. Since dicalcin binds to ZPC, and RCA-I solely reacts with the oligosaccharides of ZPC, dicalcin regulates the oligosaccharide distribution pattern within the ZP through its binding to ZPC (Figure 1). This regulation can be explained by two scenarios: one is the ‘glycoprotein’ model and the other is the ‘ZP structure’ model, although these two models are not mutually exclusive. In the ‘glycoprotein’ model, dicalcin binds to ZPC and induces an allosteric conformational change in ZPC, increasing the reaction activity of the ZP. In the ‘ZP structure’ model, dicalcin binds to ZPC and ZPB and induces changes in the three-dimensional structure of the ZP filaments, increasing the accessibility of RCA-I through the VE. Our previous study showed that both scenarios are applicable. In the first scenario, both solid- and liquid-phase assays revealed that dicalcin-bound ZPC has a greater capability to bind to RCA-I (Figure 2). In the second model, we examined *in vivo* labeling of the ZP by extracellularly-applied fluorescent dye following dicalcin treatment and found that the labeling efficiency of the ZP increased markedly in every ZP protein, not solely in ZPC, which indicates that dicalcin-binding to ZPC and ZPB causes changes in the three-dimensional structure of the entire ZP framework (Figure 3). More specifically, the observed increase in RCA-I reactivity may also involve the geometrical arrangement of ZPC whereby the ZPC molecules reside in close proximity and form the clusters of their glycans. Because a lectin generally exhibits a high affinity for glycans in clusters, this arrangement could account for a dicalcin-dependent increase in RCA-I reactivity of the ZP. Dicalcin-dependent change in the RCA-I reactivity of the ZP is unique because there are no reported examples of molecules that change the interactive affinity between proteins and carbohydrates. In future, it would be of interest to investigate the variation in the degree of the exposure of oligosaccharides on ZPC in the presence of dicalcin.

## 7. Biological Significance of Dicalcin

Our previous study showed that dicalcin-dependent changes in the distribution pattern of the RCA-I ligand correlate with the fertilization rate. Indeed, changes in the lectin staining pattern of the ZP in human oocytes have been shown to correlate with the fertilization failure [49]. These results implied that the distribution pattern of oligosaccharides within the egg-coating envelope is functionally involved with the fertilization success, which is, at least in part, regulated by dicalcin. The true biological benefit of dicalcin-induced suppression of fertilization unfortunately remains unknown at present. However, several mechanisms are known to impede fertilization, for example, inhibition of fertilization by oviductin [90], osteopontin [91], and glycodelin-A [61]. There is also a female sperm reservoir in the mammalian oviduct in which the more competent sperm are arranged (for a review see [92]), which suggests that sperm-selection mechanisms may be involved in ensuring that only higher competent sperm can reach the egg plasma membrane [93]. On the basis of this consideration, we assume that dicalcin binds to gp41 and regulates the properties of the ZP, forming a functional barrier that makes it difficult for sperm to reach the egg plasma membrane, which may favor selection of more competent sperm.

## 8. Future Perspectives

As discussed above, many carbohydrates and carbohydrate-recognizing proteins affect molecular processes involved in the success of fertilization. These variably complementary systems may act in a hierarchical and redundant manner to secure the survival of animals, and may contribute to establish a species-restricted sperm-ZP interaction. Despite extensive efforts to elucidate the molecular mechanisms of carbohydrate-mediated events in the female reproductive tract, our current understanding is still incomplete. Further investigation of the biological processes within the female reproductive tract is apparently necessary and reproductive research should remain a high priority to address the need for better understanding of fertilization, and the development of safe, reliable, and reversible birth-control methods. As highlighted in this review, dicalcin regulates the distribution pattern of carbohydrates within the ZP, affecting the fertilization rate. This evokes the interesting possibility that the probability of successful interaction between gametes may depend on the three-dimensional structure of the ZP filaments and the geometrical configuration of their carbohydrates (e.g*.*, cluster pattern and/or proper arrangement), but not solely on the composition of the carbohydrates. This concept is unique and worthy of further investigation. In addition, the potential similarity between the protein-carbohydrate interactions of reproductive cells and those of immune cells (or between immune cells and pathogens) has been pointed out [94]. It would be of interest to examine potential similar action by dicalcin in the immune system.

## Abbreviations

αGal: α-galactose
βGal: β-galactose
GlcNAc: *N*-acetylglucosamine
GalNAc: *N*-acetylgalactosamine
NeuAc: *N*-acetylneuraminic acid
